# Variation across Romania in the health impact of increasing tobacco taxation

**DOI:** 10.1093/eurpub/cky180

**Published:** 2018-10-29

**Authors:** Magdalena Ciobanu, Ionel Iosif, Cristian Calomfirescu, Lacramioara Brinduse, David Stuckler, Aaron Reeves, Andrew Snell, Kristina Mauer-Stender, Bente Mikkelsen, Alexandra Cucu

**Affiliations:** 1 Institutul National de Pneumoftiziologie “Marius Nasta” Bucuresti, Romania; 2 Institutul National de Sanatate Publica, Bucuresti, Romania; 3 Department of Social and Political Sciences, Università Bocconi, Milan, Italy; 4 Department of Social Policy and Intervention, University of Oxford, Oxford, England; 5 World Health Organisation Regional Office for Europe, Copenhagen, Denmark

## Abstract

**Background:**

Tobacco is the leading preventable cause of death globally and tobacco taxation is a cost-effective method of reducing tobacco use in countries and increasing revenue. However, without adequate enforcement some argue the risk of increasing illicit trade in cheap tobacco makes taxation ineffective. We explore this by testing sub-national variations in the impact of tobacco tax increases from 2009 to 2011, on seven smoking-related diseases in adults in Romania, to see if regions that are prone to cigarette smuggling due to bordering other countries see less benefit.

**Method:**

We use a pragmatic natural experiment study approach to analyse the study period 2009–15. Findings from hospital episodes data relating to smoking-attributable diseases are analysed for six regional subgroups which are compared according to border characteristics with other countries.

**Results:**

At a national level smoking-attributable diseases reduced over the study period especially around the tax increase years, with asthma showing the most significant decline. Sub-nationally there was no statistically significant correlation in variations between central regions and those bordering other countries.

**Conclusion:**

There is a reassuring decline in hospitalizations for smoking-related diseases associated with the tax increases, and no sub-national association with smuggling risk measured by variation in the size of this effect and regions that border other countries. More comprehensive and progressive tobacco control in Romania should be implemented in line with the WHO Framework Convention for Tobacco Control.

## Introduction

The tobacco epidemic is one of the biggest public health threats the world has ever faced, killing more than 7 million people a year.^1^ Significantly increasing the excise tax on tobacco products with the effect of an increase price to consumer is an effective tobacco use reduction tool in all ages.^2–4^

Although there is some evidence of a beneficial impact of tobacco taxation on health outcomes, primarily relating to the incidence of cancers,^5^ there remains some debate as to whether this mechanism alone is enough to improve population health improvement. This might be due to the need for a more comprehensive approach to tobacco control alongside taxation, or the difficulty in testing attributable impact with so many potential confounding influences.

Romania sharply increased its tobacco excise taxes by 28% in 2009 and by 16% in 2010. Consequently consumers paid 52% more for one pack of cigarettes in 2010 than in 2009, and 17% more in 2011 than in 2010. However, since 2011, the annual increase in excise tax has been less than 5%. No other major tobacco control initiatives took place around this period, nor were there any major control initiatives in other associated risk factors, such as alcohol or nutrition.

According to three consistent national school-based prevalence surveys that span this period,^6^ the proportion of children aged 13–15 years that smoked every day, decreased over the three surveys since 2004, with a significant decrease in 2014 compared with 2009. There are no equivalent surveys spanning this period for adult consumption. While this demonstrates a beneficial impact, there is no national evaluation of the health impacts of taxation, or how the reach of the national policy might have varied across Romania.

According to tobacco industry studies,^7^ in 2010 smuggling and illicit trade in cigarettes increased from less than 15% of total tobacco market in 2008 and 2009, to almost 35% in 2010. The scale of illicit trade varies across the country, and is greater in the counties that border other countries.^7^^,^^8^

This study uses natural experiment study techniques to evaluate the impact of Romania’s tobacco excise hikes on hospitalization rates in adults due to smoking-related diseases in Romania, and whether this impact varied across the country. A number of studies^9^ have demonstrated, decreases in hospital admissions for heart attacks, respiratory symptoms and hospitalizations for asthma and chronic bronchitis, after comprehensive smoke-free legislation was enacted, and some show a variation in impact sub-nationally, where implementation of smoke-free laws varied.^10^

This study’s hypothesis is that the excise hikes in Romania were a discrete tobacco control intervention that will beneficially impact smoking-related diseases and reduce the hospitalizations from them, and that sub-national regions that border other countries in Romania will witness a lesser impact from the hikes than central regions, owing to higher rates of smuggling in of cheap cigarettes.

## Methods

The intervention is the sharp increase of tobacco products excise in 2009 and 2010. The primary study period is 2009–15, as 2009 is the farthest back that there is comparable data available on the health outcomes. The study compares populations living in the central region of Romania where smuggling is reported as low (Intervention Group—IG with 23 counties) with border-regions where smuggling is reported^11^ as high (Control Group—CG with 19 counties).

Romania has land borders with European Union Member States (Hungary and Bulgaria) and with non-European Union countries (Moldovia, Ukraine and Serbia). The pack price of cigarettes is cheaper in the non-EU countries (e.g. in Moldova, in 2009–10, the price of one pack of cigarettes was four times cheaper than in Romania, according to mass media investigations^12^) which is a potential driver for increased smuggling in the bordering counties. The CG was also divided into subgroups according to bordering EU countries vs. non-EU countries. As the distance from the border increases through the centre of the country, the trade in illicit tobacco products would be expected to decrease. Table 1 illustrates these various groups.

**Tabel 1:** Distribution of the 41 Romanian counties plus the capital in the regional groups, according to their proximity to the border (border with a European Union country or with a non-European Union country)

Group	Region	Counties	Border
Control	SW = South-West	TM + CS + MH	Non-EU (Serbia)
	N = North	SM + MM + SV + BT	Non-EU (Ukraine)
	E = East	IS + VS + GL + TL	Non-EU (Moldova)
	W = West	BH + AR	EU (Hungary)
	S = South	DJ + OT + TR + GR + CL + CT	EU (Bulgaria)
Intervention	Centre = rest of the country	22 counties and Bucharest	No border

Data on the intervention—cigarettes total excise levels and the price of cigarettes (as of 1 July each year)—was sourced from the Ministry of Finance.^13^

Health outcomes include county-level hospitalization rates in adults (number of hospitalizations in each county per 100 000 inhabitants) for seven smoking-attributable diseases—ischaemic heart disease, stroke, chronic obstructive lung disease, asthma, tuberculosis, lung cancer and all other cancers. These data were sourced from National Institute of Public Health (hospitalizations) and National Institute of Statistics (population). We measured rates of hospitalization at the county, region and national level (as the average rate of the county hospitalized morbidity rates).

Confounders that were considered in the analysis were migration, age, gender, region of residence, health education campaigns and smoking cessation support. There were no significant differences between IG and CG for any of these characteristics.

We used the Pearson bivariate correlation to analyse the correlation between rates of hospitalization and tobacco excise tax. Linear and logistic regressions were used to study the relationship between the evolution of the rates of hospitalizations for each disease and the location of the county (IG, CG and subgroups). We developed time-series multivariate regression models for hospitalised rates by diseases, accounting for repeated observations across years, at the national level and by groups and subgroups. Mean rates of hospitalizations per region were calculated and a level of significance of *P* < 0.05 was used. The study did not use a sample representative at national and sub-national level; it was conducted using all available data about hospitalizations for the seven diseases, from 2009 to 2015.

All analyses were conducted by using SPSS 23.0.

## Results

**Figure 1:**
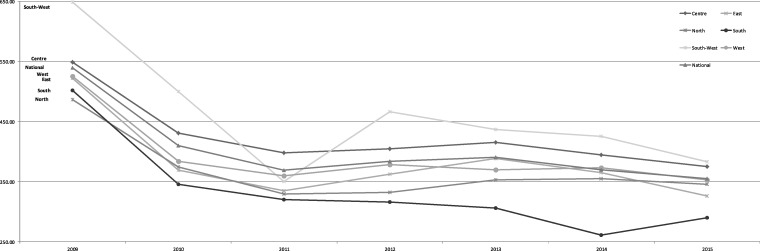
Trends in the mean rate of hospitalizations for all studied smoking-related diseases, at national and regional level, over the study period. No significant differences (*P* values > 0.01)

Hospitalization rates for the seven smoking-attributable diseases combined decreased nationally overall during the study period. However, when considering the diseases separately, the only statistically significant decline was seen in asthma. The decline in all diseases combined was steeper in the period 2009–11—the period covering the steep tax increases. Slower rates of decline and increases in South-West and East subgroups were witnessed between 2011 and 2013. After 2013, the trend was again consistently declining (figure 1). However, these sub-national variations were not statistically significant.

**Figure 2:**
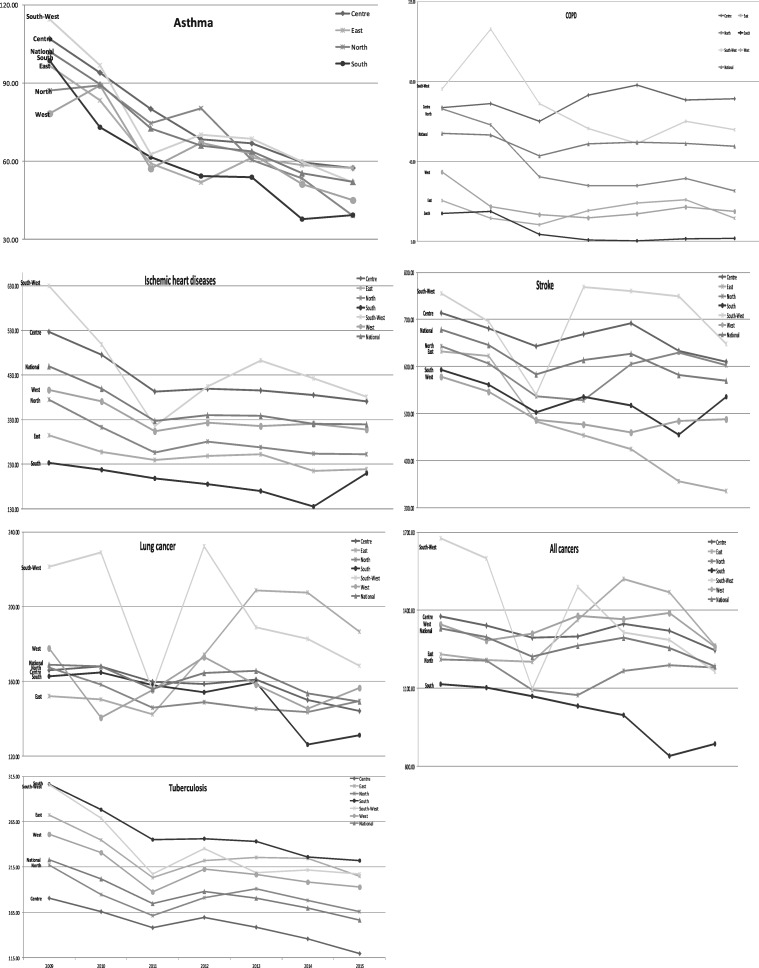
Trends in the mean rate of hospitalizations per disease and region

The trends in each disease in the six regional subgroups are illustrated in figure 2. Using a regression panel model, no statistically significant correlation was found between year-on-year changes in the mean rates of hospitalizations for any of the diseases and the border-characteristic of the groups—bordering vs. central and EU border vs. non-EU border.

When looking across the seven diseases separately, the steeper decline witnessed in all diseases combined during the period of tax hikes (2009–11) appears to be most prominent in asthma, ischaemic heart disease, stroke and Tuberculosis (TB).

From 2009 to 2010, there was a highly significant (*P* < 0.001) decrease in the mean rate of hospitalizations for TB in the Centre and North subgroups vs. a significant (*P* < 0.05) decrease in the East, South and South-West subgroups. This variation between subgroups does not demonstrate a difference between EU and non-EU border characteristics.

After a steep decrease in hospitalizations for ischaemic heart disease and stroke in 2009–11, the rate of decrease slows across most subgroups, and increases (though not significantly) in 2012 in South-West subgroup. This particular behaviour of South-West region is observed also for hospitalizations due to cancers.

## Discussion

There is an inevitable lag time between tobacco control interventions and their impact on smoking prevalence and related diseases in the population which will make the relatively short study period here a limitation. However, the health outcomes in this study are hospitalizations for the seven smoking-related diseases and so they will be mostly episodes of exacerbation (e.g. an asthma attack) or treatment (e.g. for cancer therapy) of existing disease, or for diagnosis of new but symptomatic disease. This is different from measuring prevalence or incidence of diseases, and so the issue of lag will be somewhat diminished. Smoking cessation or reduction in a person who is already suffering from one of the diseases measured here can quite quickly produce an improvement of the health status and of quality of life measured.

In this context, there is a reassuring trend of general decline witnessed across most of these smoking-attributable diseases that can be reasonably associated with the tobacco excise hikes. Whilst the study findings could not claim a definite causal effect, this association is further supported by the most prominent declines being around 2009–11—when cigarette price increases were greatest.

When looking at the diseases separately, it is only asthma that demonstrates a significant decrease over the whole period. This might relate to differing lead-times between reduced tobacco consumption and the impact on certain diseases. For example, an impact on asthma-related hospitalizations can be expected to be quicker than an impact on cancer-related hospitalizations as improvement in respiratory symptoms are often experienced quite soon after smoking cessation. However, all the diseases except cancers do demonstrate a general decline, and, excluding Chronic Obstructive Pulmonary Disease (COPD), a steeper decline associated with the intervention years.

When considered at the sub-national level, there is no obvious correlation between the border-characteristic of the six subgroups and the size of the impact on smoking-related diseases. This might be owing to a number of explanations: smuggling in of cheaper cigarettes might not have increased to the extent that the tobacco industry studies implied; any smuggled cigarettes might have been subsequently distributed more evenly throughout Romania; smuggled cigarettes might have been further ‘exported’ onto other EU Member States, rather than sold in Romania; or any changes in smuggling might not have actually affected tobacco consumption sub-nationally, or to an extent that was detectable. To support some of these explanations, there is international evidence that tobacco tax increases are not associated with a significant increase in illicit trade.^8^ However, to these sub-national impacts more depth primary data collection and further research might help to further inform and strengthen tobacco control in countries like Romania.

There are a number of limitations in this study. There is a lack of comparable data in the years leading up to the intervention and the study period was quite short. The data used are all secondary data, and so not collected for the purpose of the study. There will be some lag periods between reduced smoking and reduced hospitalizations, and these will differ for each of the diseases.

Other factors around this period that should be considered as potential confounders include the economic crisis in Romania that started around 2008. However, this might more reasonably be considered as a contributor rather than a confounder—it could be expected to increase the effect size of cigarette price increases creating a combined impact on reducing access to tobacco through affordability. As the intervention is associated with reduced smoking in school children aged 13–15 years old, this positive consequence could have a short- and medium-term significant impact on hospitalizations for asthma, including in adult period, explaining, even partially, the significant positive improvement of this outcome.

With these limitations, it is not possible to reliably attribute the changes observed directly to tobacco taxation. However, it is reasonable to consider the witnessed trends as an association that supports the tobacco taxation despite the perceived risk of increases in illicit trade, and supports strengthening tobacco control laws in Romania.

Beyond the two large tobacco tax excise hikes of 2009 and 2010, there were no other significant steps towards tobacco control in Romania. The evidence and international guidance, including the WHO FCTC and their MPOWER ‘best buys’ measures, are very clear in what constituents’ comprehensive tobacco control. To ensure a significant and sustainable beneficial impact, all the measures should be implemented and be progressive. If Romania had taken a more comprehensive approach then more convincing trends in beneficial impact might be demonstrable. Moving forward, Romania should prioritize implementing a tobacco control programme that is in line with the WHO FCTC.

## Funding

The Natural Experiment Study project is run by the WHO Regional Office for Europe and supported by funding from the Ministry of Health of the Russian Federation and the Ministry of Health and Medical Industry of Turkmenistan. David Stuckler is funded by a Wellcome Trust Investigator Award and ERC HRES 313590.

*Conflicts of interest*: None declared.
